# Evaluating the effects of maternal positions in childbirth: An overview of Cochrane Systematic Reviews

**DOI:** 10.18332/ejm/142781

**Published:** 2021-12-21

**Authors:** Marion Kibuka, Amy Price, Igho Onakpoya, Stephanie Tierney, Mike Clarke

**Affiliations:** 1 Department for Continuing Education, University of Oxford, Oxford, United Kingdom; 2 Stanford Anesthesia and Informatics Media Lab, School of Medicine, Stanford University, Palo Alto, United States; 3 Centre for Evidence Based Medicine, University of Oxford, Oxford, United Kingdom; 4 Nuffield Department of Primary Care Health Sciences, University of Oxford, Oxford, United Kingdom; 5 All Ireland Hub for Trials Methodology Research, Centre for Public Health, Institute of Clinical Sciences, Queen’s University Belfast, Royal Victoria Hospital, Belfast, Ireland

**Keywords:** mode of birth, overview of Cochrane reviews, maternal position, length of labor and birth, first and second stages of labor

## Abstract

**INTRODUCTION:**

The purpose of this study is to conduct an overview of Cochrane systematic reviews (SRs) evaluating the effects of maternal positions in childbirth in order to compile existing evidence for relevant research questions that have been addressed by more than one review, to provide a succinct summary of the up-to-date evidence and to identify areas for future research.

**METHODS:**

An electronic search was conducted in the Cochrane database. Two primary outcomes were the duration of labor and birth, and operative birth. The quality of included reviews was assessed using the AMSTAR criteria, and the quality of the evidence was rated using the GRADE criteria.

**RESULTS:**

We included 3 Cochrane SRs. There was a significant mean difference (MD) found in the duration of the first stage by 1 hour and 22 minutes (MD= -1.21; 95% CI: -2.35 – -0.07, I^2^=94%) and reduction in caesarean section rates (RR=0.71; 95% CI: 0.54–0.94, I^2^=0%) in the upright birth position group compared with the horizontal. Also, there was a statistically significant difference in the duration (minutes) of the second stage of labor (MD= -6.16; 95% CI: -9.74 – -2.59, I^2^=91%) and a reduction in assisted vaginal birth rates (RR=0.75, 95% CI: 0.66–0.86, I^2^=29%) in the upright group compared with the horizontal without epidural analgesia. The quality of evidence within the reviews was very low to moderate.

**CONCLUSIONS:**

There is currently a limited body of evidence to clearly assess the benefits and risks of assuming upright positions during childbirth. The overview highlights the need for high-quality research studies, involving better definition and comprehensive assessment of the effects of squatting during childbirth.

## INTRODUCTION

The objective of this overview of Cochrane systematic reviews (SRs) was to provide a summary of the effect of upright compared with horizontal positions on maternal and fetal outcomes during childbirth among women who took or did not take epidural analgesia. An electronic search was conducted in the Cochrane database. Two primary outcomes were: 1) duration of labor and birth, and 2) operative birth. Three Cochrane SRs were included (65 trials with 18697 women). In the first stage of labor, without epidural analgesia, upright positions were significantly associated with shorter duration of labor (mean difference, MD= -1.36; 95% CI: -2.22 – -0.51) and a reduction in the number of caesarean sections (risk ratio, RR=0.71; 95% CI: 0.54–0.94) but with no significant difference in the rates of assisted vaginal births (RR=0.91; 95% CI: 0.73–1.14). In the first stage of labor among women using epidural analgesia, there were no significant differences between groups in the rates of caesarean section (RR=1.05; 95% CI: 0.83–1.32) and assisted vaginal birth (RR=1.06; 95% CI: 0.90–1.25). In the second stage of labor, among women without epidural analgesia, the upright position was associated with a significant but small reduction in the duration (minutes) of that stage of labor (MD= -6.16; 95% CI: -9.74 – -2.59) and a reduction in the number of assisted deliveries (RR=0.75; 95% CI: 0.66–0.86), but there was no significant difference in the rate of caesarean sections (RR=1.22; 95% CI: 0.81–1.81). The overall effect measure showed no significant difference in operative birth rates (RR=0.86; 95% CI: 0.70–1.07) and durations (minutes) of birth (MD=6.00: 95% CI: -37.46–49.46) between the upright and the horizontal groups during the second stage of labor when the women took epidural analgesia. The three reviews were rated as high quality using the Revised Assessment of Multiple Systematic Reviews (R-AMSTAR) criteria. The results of the SRs identified some benefits when women adopted upright positions during labor and birth. However, uncertainty remains as to whether the effectiveness of the RCTs of this overview can be replicated. The overview highlights the need for high-quality research studies, involving better definition and comprehensive assessment of the effects of squatting during childbirth. Four new research questions emerge from the overview findings:

1. How does the use of the squatting position during childbirth work in clinical practice?
2. What are the potential benefits and harms of squatting compared with other positions in the second stage of labor for low-risk women?
3. What are the perspectives of women, their partners and healthcare providers regarding the barriers to and facilitators of the use of upright/squatting positions in childbirth?
4. What are the key priorities for the development of the definition of the squatting position in childbirth among key stakeholders?

### Background

Maternal positioning affects the biomechanics and physiologic adaptions to labor. The biomechanical mechanisms of birth positioning, which are associated with pelvic dimensions, intrauterine pressure, fetal head molding and progression of fetal head angle through the birth canal, have more recently been studied^1-3^. The article of Atwood^4^ maintains the standard for the definition of the two main maternal positions during childbirth, i.e. upright and horizontal, based on the angle made by the horizontal plane and the line linking the midpoints of the third and fifty lumbar vertebrae. When the spine is vertical and >45 degrees, the position is considered upright, and horizontal when <45 degrees^4^.

Upright positions offer a number of advantages. Gravity can promote the descent of the fetus. Pelvic outlet dimensions are increased reducing the likelihood of labor dystocia^3,5,6^. Maintaining fetal flexion with the smallest possible cross-section passing through the birth canal, leads to optimal fetal posterior-anterior positioning. Furthermore, hip flexion such as in the squatting position significantly increases the fetal head angle of progression through the pelvic axis and the soft tissues of the cervix and pelvic floor, contributing to a spontaneous vaginal birth^1^. Uterine contractions are generally stronger and more efficient in effacing and dilating the cervix^2,7^, resulting in a shorter duration of labor and birth^8,9^. An upright position also is beneficial to the mother’s cardiac output, which normally increases during labor and promotes good fetal circulation^10^.

According to good quality worldwide scientific evidence, the horizontal position at the time of labor and birth increases the occurrence of caesarean sections, instrumented vaginal births, episiotomies, and abnormal fetal heart rate^8,9^. However, the certainty of evidence is unclear, since frequent changes in position relieve fatigue, increase comfort and improve maternal blood circulation. Therefore, it has been recommended that health providers should not impose a birth position but rather encourage free choice of position, including upright ones that are most comfortable for the woman^8^.

The effect of hip extension and restrictive movement of the sacrum in horizontal compared with hip flection and flexible movement of the sacrum when upright during childbirth, require further investigation^11,12^. At this stage, it is timely and crucial to bring together overview summaries of all relevant Cochrane systematic reviews of randomized controlled trials to evaluate the effects of upright compared with horizontal positions during labor and birth on birth outcomes. An overview of systematic reviews involves the identification, retrieval, assessment and syntheses of the evidence from multiple systematic reviews^13^. Campbell et al.^14^ have pointed out that an overview of the existing evidence on a complex intervention should be a starting point for defining the extent of the clinical problem, assessing the benefits and harms of the intervention, identifying gaps in research and informing the developmental planning stage for the new intervention. This view has been supported by other authors^15-17^. The overarching review question this study addresses is: ‘What is known about the effects of maternal positions during childbirth?’.

### Study objectives

To compile a summary of the best available evidence from Cochrane systematic reviews (SRs) of randomized controlled trials (RCTs) on the benefits and harms of upright versus horizontal positions on the mode of birth, and duration of the first and second stages of labor.

## METHODS

### Criteria for inclusion of reviews

*Types of study that were included*

The overview included RCT and quasi-RCT studies that had evaluated randomized trials, since they were deemed to be the most rigorous and transparent studies. Unpublished reviews and any reviews that had evaluated non-randomized studies were excluded.

*Type of participant*

The inclusion criteria defined the eligible population as pregnant women of any parity (i.e. primigravida, multigravida, or mixed) who had experienced spontaneous or induced labor at the full-term of their pregnancies (>37 weeks’ gestational age), using any type of analgesia.

*Type of intervention*

The type of intervention was the position or positions assumed by women in the first and second stages of labor. The positions assumed in the first and/or second stages of labor can be broadly categorized as being either upright or recumbent.

Upright has been defined as ‘erect or vertical’ positions that are flexible sacrum positions, where the coccyx is free to move, occur at a rotation of 15.7° of the coccyx with a widening of the pubic symphysis of 3 mm, which appear to be more beneficial for the mother’s pelvis^12^. It has been urged that during childbirth, the coccyx rotates outwards in the sagittal plane due to the force of the fetus on the structure, thereby opening the pelvic outlet. Therefore, the positions considered upright in the experimental group included:

- Sitting
- Standing
- Walking
- Kneeling
- Squatting, e.g. unsupported/deep or supported by equipment, supported by companion or not
- All fours (hands and knees) as defined by the authors.

*Type of comparison*

In contrast, horizontal positions have been classified as non-flexible sacrum positions, where the coccyx movement is restricted, occur at a rotation of 3.6° of the coccyx and with a widening of 6 mm of the pubic symphysis^12^. The positions considered horizontal in the comparison group were as follows:

- Supine
- Lithotomy
- Semi-recumbent or recumbent
- Lateral
- Dorsal
- Bed care
- Trendelenburg’s positions.

*Types of outcomes*

Three main maternal outcomes included:

1. Duration of labor (i.e. first stage of labor)
2. Duration of birth period (i.e. second stage of labor)
3. Mode of birth (operative birth, defined as a sum of caesarean sections and assisted birth or subgroup of each of these).

### Literature search

The Cochrane Database of Systematic Reviews (CDSR) and Archie (the Cochrane information management system) were searched on 4 March 2020 for relevant reviews that had been published up to that date. No restrictions on language, date of publication or geographical area were imposed, but Cochrane SRs are published primarily in English.

A sensitive search strategy for the CDSR database was developed through a combination of index terms and text keywords that were relevant to the condition, intervention and outcome. Free text keywords included: [upright OR position OR supine] and [first OR second OR stage OR labor]. The search was limited to finding the search terms in the title, abstract or keywords of the reviews.

### Data extraction

The data were extracted independently using predefined extracted worksheets, and cross-checked for accuracy and completeness. The data extraction process was then verified and information was extracted from each SR. This included:

- General information, e.g. first author’s name, contact details;
- Number of included studies, details of the participants and search strategies;
- Inclusion and exclusion criteria and methodological quality assessment;
- Participant information;
- Outcomes; and
- Results.

### Quality of included SRs and body of evidence

It was intended to assess two aspects of quality for the included reviews. These included: the quality of evidence within the SRs (primary studies included in the SRs) and the quality of the SRs themselves. Quality assessments were performed for each review using the Revised Assessment of Multiple Systematic Reviews (R-AMSTAR) parameters^18,19^. Furthermore, data were extracted on the overall body of evidence using the numerical guides of the Grading of Recommendation, Assessment, Development and Evaluation (GRADE) tool^20^ to provide the overall quality of evidence of specified outcomes. The quality of included reviews was independently assessed as was the overall quality across the included primary studies; the processes of this section were verified and discussed.

### Timing and effect measures

The characteristics of included SRs were summarized by tabulating the proportions of relative measures of effect (risk ratio, RR) with 95% confidence interval (CI) for dichotomous outcomes, or by reporting the mean difference (MD) with 95% CI for continuous outcomes. The results are presented as reported in each of the included Cochrane SRs, without additional analysis of the data.

### Data analysis

All the included reviews carried out statistical analysis using the Review Manager software^21^. Fixed-effect meta-analysis for combining data was used if it was reasonable to assume that studies were estimating the same underlying treatment effect, i.e. where trials that were examining the same intervention, and the trials’ populations and methods were judged to be sufficiently similar.

Where clinical heterogeneity was sufficient to expect that the underlying treatment effects differed between trials, or if substantial statistical heterogeneity was detected, random-effects meta-analysis was used to produce an overall summary if an average treatment effect across trials was considered clinically meaningful. The random-effects summary was treated as the average range of possible treatment effects and the results were presented as the average treatment effect with 95% CI, and the estimates of tau^2^; and I^2^;.

## RESULTS

### Results of the overview of Cochrane SRs

*Management of the overview of SR data*

**Figure 1:**
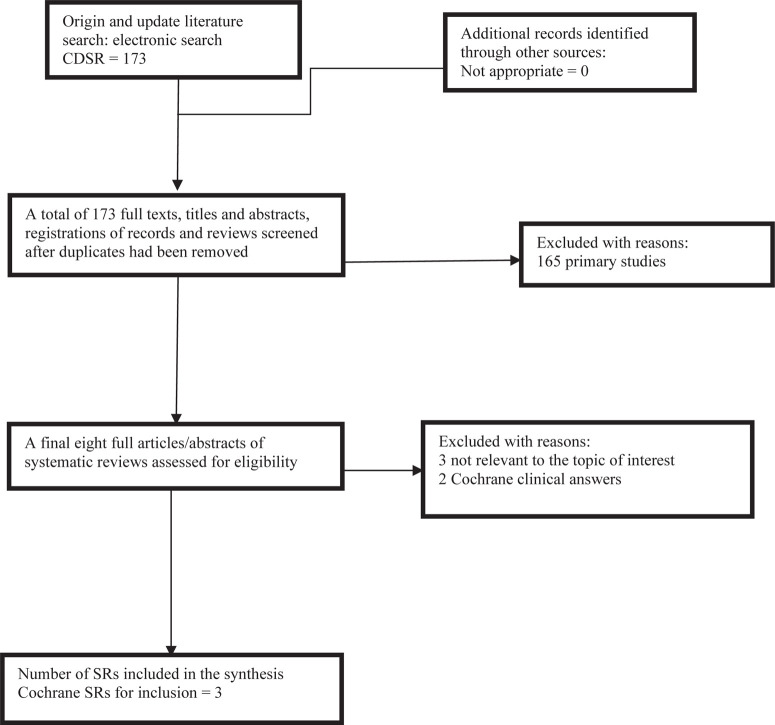
Flowchart showing the inclusion of SRs that assessed the effects of upright versus horizontal positions in child birth on maternal and fetal outcomes

The literature search identified a total of 173 non-duplicate citations, of which eight SRs were assessed in full. Three met the eligibility criteria and five SRs were excluded (three because they were not relevant to the topic and two that were Cochrane clinical answers). Figure 1 shows the process by which the inclusion of SRs was decided. The characteristics of the reviews that were included are summarized in Table 1.

**Table 1:** Characteristics of included SRs

*Review ID*	*Date of last search; Date of latest publication; Evaluation*	*Number of trials and women included*	*Study’s countries of origin*	*Inclusion and exclusion criteria*	*Intervention and comparison*	*Outcome measurements*	*Obstetric condition*	*R-AMSTAR & GRADE quality of the evidence*
Lawrence et al.^9^	Search 13/1/2013 Published 6/2013 Out of date	25 trials with 5218 women	13 countries Australia, Brazil, Finland, France, Hong Kong, Iran, Japan, Sweden, Taiwan, Thailand, United Kingdom, United States of America.	Studies: RCTs,quasi-randomization,cluster randomization. Population: any parity receiving or not receiving epidural analgesia. Condition: first stage of labor. Timescale: Nopublication of trial limit.	Interventions: Sitting Standing Walking Kneeling Squatting All fours (hands and knees) Comparisons: Semi-recumbent Lateral Supine Dorsal Bed care	*Primary outcomes* Maternal outcomes: Duration of first stage Mode of birth (spontaneous vaginal birth, operative and caesarean births) Maternal satisfactory Fetal outcomes Fetal distress Need for ventilation *Secondary outcomes* Maternal outcomes: Pain Use of analgesics Duration of second stage Augmentation of labor Artificial rupture of membranes Hypotension Blood loss >500 mL Perineal trauma Fetal/neonatal outcomes: Apgar scores Admission to NICU Perinatal death	First stage of labor with and without epidural analgesia	43 Not reported
Gupta et al.^8^	Search 30/11/2016 Published 2017 Up to date	32 trials with 9015 women	17 countries Brazil, China, Finland, France, Hong Kong, India, Iran, New Zealand, Nova Scotia, Pakistan, Palestine, Iran, South Africa, Sweden, Thailand, Turkey, United Kingdom.	Studies: RCTs, quasi-randomization, cluster randomization. Population: any parity not epidural analgesia. Condition: second stage of labor. Outcomes: any outcome reported in the review. Timescale: No publication of trial limit.	Interventions: Sitting (obstetric chair/stool) Kneeling (all fours) Squatting (unaided or using squatting bars) Squatting (aided with birth cushion) Comparisons: Lateral (Sim’s) position Dorsal Semi-recumbent (trunk tilted forwards up to 30Ð to the horizontal) Lithotomy position Trendelenburg’s position	Primary outcomes Maternal outcome: Duration of second stage of labor Secondary outcomes Maternal outcomes: Pain Use of any analgesia or anesthesia. Assisted birth. Caesarean birth Episiotomy Second-degree tears Third- and fourth- degree tears Blood loss >500 mL Need for blood transfusion. Manual removal of placenta. Shoulder dystocia Urinary incontinence Fecal incontinence Fetal outcome: Abnormal FHR patterns Neonatal outcomes: Admission to NICU Perinatal death	Second stage of labor without epidural analgesia	44 Moderate to very low
Walker et al.^22^	Search 5/6/2018 Published 2018 Up to date	8 trials with 4464 women	3 Countries France (1 trial) United Kingdom (5 trials) Spain (2 trials)	Studies: RCTs, quasi-randomization. Population: any parity receiving epidural analgesia. Condition: second stage of labor. Timescale: No publication of trial limit.	Interventions: Sitting (on a bed) Sitting Squatting (unaided or using squatting bars) Squatting (aided with birth cushion) Semi-recumbent (we classed this as an upright position if the main axis of the body [chest and abdomen] was 45° or more from the horizontal) Kneeling Walking Comparisons: Lithotomy position Lateral position Trendelenburg’s position Knee-elbow (all fours) position Semi-recumbent (we classed this as a recumbent position if the main axis of the body [chest and abdomen] was less than 45° from the horizontal).	Primary outcomes Maternal outcomes: Operative birth Duration of second Stage Secondary outcomes Maternal outcomes: Caesarean birth Assisted birth Trauma to birth canal Blood loss > 500 mL Prolonged second stage > 60 minutes Maternal experience Baby outcomes: Abnormal FHR patterns (FHR fetal heart rate) Apgar scores <7 at 5 min Low cord pH <7.1 Admission to NICU Need for ventilation Perinatal death	Second stage of labor with epidural analgesia	43 High to very low

*Type of study included in the overview of reviews*

The SRs that were included in the overview comprise 65 RCTs, of which 50 trials (77%) were of parallel group design, while the remaining 15 trials (23%) were quasi-randomized studies. No cluster randomized studies were included in the SRs.

*Description of participants included in the overview of reviews*

The SRs involved a total of 18697 women^8,9,22^, with sample sizes ranging from 66 to 3236 participants in the studies. Of the 65 studies that were included in the SRs, 30 trials considered only primiparous women, four investigated only multiparous women, and 31 studied women of mixed parity. All trials included women who were either experiencing singleton pregnancies at term (>37 weeks of gestation) or who were at >34 weeks of gestation.

*Geographical settings and dates*

The studies included in the SRs were conducted in 23 countries. They involved women from low-middle to high-income countries, mainly in hospital settings, over a period of 56 years, and had been published between 1963 and 2016.

*Types of interventions and controls*

A broad range of classifications of upright and horizontal positions was identified in the included SRs. One defined the upright position as requiring the angle of the spine to be >30 degrees^8^ while another cited a 45° angle^22^ and the third provided no information on the angle of the spine^9^. Furthermore, there were discrepancies in the descriptions of different positions (e.g. the all fours position was considered an upright position in two SRs^8,9^, while in one SR it was considered horizontal^22^). Two included SRs measured the direction and magnitude of the effect through subgroup analysis by position type. These included sitting, walking, squatting, kneeling, all fours and squatting on cushion, chair or stool, compared with horizontal^10^.

*Quality of the trials that were included within SRs*

The three SRs also provided a rating of methodological quality for the included trials by application of the Cochrane risk of bias tool. The specific details of the assessments of risk of bias that were reported in the included SRs are summarized in Table 2. In all the included studies, it was not possible to blind the intervention from the participants, healthcare personnel or outcome assessors. Also, in all three SRs, poor reporting of random sequence generation, allocation concealment and other parameters of Cochrane risk of bias in primary studies were stated. Therefore, the quality of evidence within the studies included in the SRs was variable (Table 2).

**Table 2:** Risk of bias (RoB) summary: review authors’ judgements regarding each RoB item for studies within the reviews

*Item*	*Gupta et al.^8^ (32 trials)*			*Walker et al.^22^ (8 trials)*			*Lawrence et al.^9^ (25 trials)*		
	*LRB*	*URB*	*HRB*	*LRB*	*URB*	*HRB*	*LRB*	*URB*	*HRB*
Random sequence allocation	8	15	9	6	2	0	5	14	6
Allocation concealment	1	22	9	2	6	0	8	12	5
Performance bias	0	1	31	0	0	8	0	1	24
Detection bias	0	28	4	0	0	8	0	1	24
Attrition bias	21	3	8	4	2	2	16	4	5
Reporting bias	23	1	8	1	5	2	0	19	6
Other bias	32	0	0	1	4	5	-	-	-

LRB: low risk of bias. HRB: high risk of bias. URB: unclear risk of bias.

*Quality assessment of the included SRs using the AMSTAR tool*

Based on the R-AMSTAR criteria, the three included SRs were rated high quality (i.e. they had an R-AMSTAR score of ≥40)^8,9,22^. Table 1 gives the R-AMSTAR scores of each individual SR. The authors of one SR that had investigated eight trials did not assess the work for publication bias because the precision of studies that were included in the meta-analysis would be biased, since fewer than 10 effect sizes were analysed^23-25^. All SRs contained tables that showed information regarding the studies that had been included and excluded.

*Quality of the evidence included in the reviews*

In two SRs^8,22^, the GRADE approach was applied to grade study quality, although one SR did not present sufficient information to assess quality of evidence externally^9^. The GRADE assessments that are presented in the summary of findings table from two of the included SRs^8,22^ show judgements made by the SR authors. The quality of the evidence for outcomes that were reported from studies in the SRs varied widely, as shown by the GRADE assessments in each SR. The overall quality of evidence within the SRs ranged from moderate to very low.

*Summary of the results of the overview of the included SRs*

This overview reports the pre-specified primary maternal outcomes as reported in the included SRs. The summary of findings table (SoF) (Table 3) shows the main outcomes from the three included SRs that assessed the use of an upright compared with a horizontal position during labor and birth when the mothers took or did not take epidural analgesia. The table shows the number of trials that were included in the meta-analysis, the number of women involved, the RR with 95% CI, the I^2^ measure, which was used for heterogeneity with tau^2^ and p values, and the quality rating of the most important outcomes.

**Table 3:** Summary of findings for the main comparisonsa,b of any upright positions compared with horizontal during the second stage of labor with and without epidural analgesia^8,9,22^

*Review ID and Table number*	*Outcomes*	Anticipated absolute effects*		*Relative effects of upright vs supine*	*Number of participants and studies*	*Quality of the evidence (GRADE)*	*Comments*
		*Risk with supine position*	*Risk with any upright position*	*(RR or MD with 95% CI and measure of heterogeneity)*			
**Duration of first stage of labor**							
**CL1 1.1**	Duration of first stage of labor without epidural analgesia			The mean duration of first stage of labor was 1.22 hours shorter in the upright position (2.13 to 0.31 hours shorter) I^2^=93%, tau^2^=3.42, p<0.00001	2502 15 RCTs	Not reported	Including primigravida and multigravida women Favors upright
**CL2 1.2**	Duration of first stage of labor with epidural analgesia			No significant difference in the mean duration of first stage between the two groups average MD= -3.71 hours (-9.37-1.94) MD= 2.35 minutes higher (-15.22-19.91) I^2^=0%, tau^2^=0.00, p=0.44	204 2 RCTs	Not reported	No significant difference
**Duration of second stage of labor**							
**CL1 2.1**	Duration of second stage of labor without epidural analgesia			No significant difference in the mean duration of second stage between the two groups MD= -3.71 hours (-9.37-1.94) I^2^=73%, tau^2^=51.97, p=0.00026		Not reported	
**CG3 2.2**	Duration of second stage of labor without epidural analgesia			The mean duration of second stage of labor was 6.16 minutes shorter in the upright position (9.74 minutes shorter to 2.59 minutes shorter) I^2^=98%, tau^2^=1404.42, p<0.00001		Very low ⊕⊖⊖⊖	Including multigravida and primigravida women Favors upright
**CW4 2.3**	Duration of second stage of labor with epidural analgesia			The mean duration of second stage labor across control groups ranged from 52.06 minutes to 124.3 minutes. MD= 6.00 minutes higher (37.46 lower to 49.46 higher) I^2^=91%, tau^2^=56.35, p<0.00001		Very low ⊕⊖⊖⊖	No significant difference
**CW4 2.4**	Duration of pushing phase >60 minutes			MD= -16.37 (-24.55 – -8.19)	199 1 RCT	Not reported	Favors upright
**Mode of birth: operative birth (caesarean section or assisted vaginal birth)**							
**CW4 3.1**	Operative birth with epidural analgesia	Study population 554/1000	476/1000 (382-592)	RR=0.86 (0.70-1.07) I^2^=49%, tau^2^=0.00, p=0.16	4316 8 RCTs	Low ⊕⊕⊖⊖	No significant difference
**Mode of birth: assisted birth**							
**CL1 4.1**	Assisted birth without epidural analgesia			RR=0.91 (0.73-1.14) I^2^=21%, tau^2^=0.00, p=0.24	2519 13 RCTs	Not reported	No significant difference
**CL2 4.2**	Assisted birth with epidural analgesia			RR=1.06 (0.90-1.25) I^2^= 0%, tau^2^=0.00, p=0.58	1568 6 RCTs	Not reported	No significant difference
**CG3 4.3**	Assisted birth without epidural analgesia	Study population 128/1000	96/1000 (84-110)	RR=0.75 (0.66-0.86) I^2^=29%, tau^2^=0.00, p=0.11	6481 21 RCTs	Moderate ⊕⊕⊕⊖	Favors upright
**CW4 4.3**	Assisted birth with epidural analgesia	Study population 468/1000	421/1000 (337-524)	RR=0.90 (0.72-1.12) I^2^=69%, tau^2^=0.00, p=0.002	4316 8 RCTs	Very low ⊕⊖⊖⊖	No significant difference
**Mode of birth: caesarean section**							
**CL1 5.1**	Caesarean section without epidural analgesia			RR=0.71 (0.54-0.94) I^2^=42%, tau^2^=1.47, p=0.19	2682 14 RCTs	Not reported	Favors upright
**CL2 5.2**	Caesarean section with epidural analgesia			RR=1.05 (0.83-1.32) I^2^=17%, tau^2^=0.00, p=0.31	1566 6 RCTs	Not reported	No significant difference
**CG3 5.3**	Caesarean section without epidural analgesia	Study population 14/1000	18/1000 (12-26)	RR=1.22 (0.81-1.81) I^2^=0%, tau^2^=0.00, p=0.49	5439 16 RCTs	Low ⊕⊕⊖⊖	No significant difference
**CW4 5.4**	Caesarean section with epidural analgesia	Study population 86/1000	81/1000 (52-125)	RR=0.94 (0.61-1.46) I^2^=69%, tau^2^=0.13, p=0.07	4316 8 RCTs	Very low ⊕⊖⊖⊖	No significant difference

a Any upright compared to supine position for the second stage of labor for women without epidural anesthesia. b Patient or population: women in the second stage of labor without and with epidural anesthesia. Setting: hospital settings. Intervention: any upright position. Comparison: supine position.

* The risk in the intervention group (and its 95% confidence interval) is based on the assumed risk in the comparison group and the relative effect of the intervention (and its 95% CI). CI: confidence interval. RR: risk ratio. MD: mean difference.

The summary of results presented in Table 3 are in the following format using abbreviations for each comparison:

- Comparison 1 is called CL1, in which C stands for comparison and L for Lawrence. CL1 presents the analyses by Lawrence et al.^9^ who evaluated maternal positions in the first stage of labor when epidural analgesia was not used.
- Comparison 2 is called CL2, in which, again, C stands for comparison and L for Lawrence. CL2 presents the analyses by Lawrence et al.^9^ who evaluated maternal positions in the first stage of labor when mothers used epidural analgesia.
- Comparison 3 is called CG3, in which C stands for comparison and G for Gupta. CG3 presents analyses that were reported by Gupta et al.^8^ who evaluated maternal positions in the second stage of labor without epidural analgesia.
- Comparison 4 is called CW4, in which C is for comparison and W for Walker. CW4 presents analyses reported by Walker et al.^22^ who evaluated maternal positions during the second stage of labor when epidural analgesia was used.

### Primary maternal outcomes

*Duration of the first stage of labor*

The duration of the first stage of labor was reported in one SR that was included in this overview^9^. Fifteen RCTs that included women who did not use epidural analgesia found that the period of the first stage was one hour and 22 minutes shorter for the upright group than for those in recumbence and this difference was statistically significant (MD= -1.36; 95% CI: -2.22–0.51, I^2^=93%, tau^2^=2.39, p<0.00001; 2503 women; quality of evidence not reported). However, RCTs pooled for the duration of first stage of labor among women using epidural analgesia did not report this outcome.

*Duration of the second stage of labor*

The duration of the second stage of labor was reported in all three SRs that were included in this overview^8,9,22^. In one SR^9^, nine RCTs reported that they had found no significant difference in the mean duration of the second stage of labor between upright and horizontal groups of women, when these positions were maintained during the first stage of labor without epidural analgesia (MD= -3.71, 95% CI: -9.37–1.94, I^2^=73%, tau^2^=51.97, p= 0.00029; 2077 women; certainty of evidence not reported). Similarly, two RCTs found no significant difference in the mean duration of the second stage of labor between the two groups of women using epidural analgesia in the first stage of labor (MD=2.35; 95% CI: -15.22–19.91, I^2^=0%, tau^2^=0.0, p= 0.44; 204 women; certainty of evidence not reported). The other two SRs investigated the effect of upright compared with horizontal positions during the second stage of labor only. They included no criteria regarding how the women had experienced the first stage of labor^8,22^. In one SR^8^, 19 RCTs reported a significantly shorter duration of the second stage of labor, by 6 minutes and 16 seconds, in the upright group compared with the horizontal among those who did not use epidural analgesia (MD= -6.16 minutes, 95% CI: -9.74 – -2.59, I^2^=91%, tau^2^=56.35, p<0.00001; 5811 women; very low certainty of evidence of effect) (Table 4).

**Table 4:** Summary findings of meta-analyses with contradicting direction of effect sizes on squatting positions in Gupta et al.^8^ Cochrane Review

*Outcome*	*Intervention (squatting vs supine)*	*Analysis (MD or RR with 95% CI; number of RCTs; level of evidence)*	*Results*
Duration of second stage of labor	Main analysis	MD= -6.16 (-9.74 – -2.59); 19; Very low	Significant
	Squatting using birth cushion	MD= -10.64 (-20.15 – -1.12); 3; Not reported	Significant
	Squatting using birth stool	MD= -0.57 (-3.83–2.68); 4; Not reported	Non-significant
	Squatting using birth chair	MD= -2.63 (-7.03–1.77); 9; Not reported	Non-significant
Assisted births	Main analysis	RR=0.75 (0.66–0.86); 21; Moderate	Significant
	Squatting using birth cushion	RR=0.50 (0.32–0.78); 2; Not reported	Significant
	Squatting using birth stool	RR=0.77 (0.58–1.01); 8; Not reported	Non-significant
	Squatting using birth chair	RR=0.91 (0.643–1.30); 8; Not reported	Non-significant

Among women who used epidural analgesia^22^, three RCTs found no significant difference in the duration (minutes) of the second stage between the groups who assumed upright versus horizontal positions (MD=6; 95% CI: -37.46–49.46; I^2^=96%, tau^2^=1404.42, p<0.00001; 456 women; very low-quality certainty of evidence), but one RCT showed a significant reduction of the duration of the prolonged second stage of labor (pushing for more than 60 minutes) when the women used upright positions compared with those who lay horizontally (MD= -16.37; 95% CI: -24.55 – -8.19; I^2^, tau^2^ and p not measurable; 199 women; certainty of evidence not reported).

### Mode of birth

*Operative birth (caesarean section or assisted vaginal birth)*

The sum of operative births (caesarean sections plus assisted vaginal births) was reported in one of the three SRs that were included in the overview^22^. Eight RCTs found no significant difference in the rates of operative births between the upright and horizontal positions in the second stage of labor among those who used epidural analgesia (RR=0.86, 95% CI: 0.70– 1.07, I^2^=78%, tau^2^=0.06, p=0.00005; 4316 women; low certainty of evidence of effect).

*Assisted vaginal birth*

Assisted vaginal birth outcomes were reported in all three included SRs^8,9,22^. Seven RCTs found no significant difference between the rates of assisted vaginal birth in both comparative groups during the first stage of labor without epidural anesthesia (RR=1.17; 95% CI: 0.88–1.57, I^2^=96%, tau^2^=1404.42, p<0.00001; 1773 women; certainty of evidence not reported) and three RCTs with epidural analgesia (RR=1.02; 95% CI: 0.86–1.20, I^2^=0.0%, tau^2^=0.00, p=0.45; certainty of evidence not reported)^9^. However, in the second stage of labor among women who did not use epidurals, overall analyses of 21 RCTs showed that rates of assisted vaginal birth were significantly reduced in the upright group compared with the horizontal (RR=0.75; 95% CI: 0.66–0.68, I^2^=29%, tau^2^=0.00, p=0.11; 6481 women; moderate certainty of evidence of effect)^8^. In contrast, during the second stage of labor with epidural analgesia, eight RCTs found no significant difference in the rates of assisted vaginal birth between upright and horizontal groups (RR=0.90; 95% CI: 0.72–1.12, I^2^=69%, tau^2^=0.00, p=0.002; very low certainty of evidence of effect)^22^.

*Caesarean section*

Overall, among women who did not use epidurals, 14 RCTs found a significant reduction in caesarean section rates among those who chose the upright compared with the horizontal position during the first stage of labor (RR=0.71; 95% CI: 0.54–0.94, I^2^=42%, tau^2^=1.47, p<0.19; 2682 women; certainty of evidence not reported), although six RCTs found no significant difference between the groups of women using epidural analgesia (RR=1.05; 95% CI: 0.83–1.32, I^2^=17%, tau^2^=0.00, p=0.31; 1566 women; certainty of evidence not reported)^9^.

Similarly, among women who chose not to take epidural analgesia during the second stage of labor, there was no significant difference between upright and horizontal groups in the rates of caesarean section in the overall analysis of 16 RCTs (RR=1.22; 95% CI: 0.81–1.81, I^2^=0%, tau^2^=0.0, p=0.49; 5439 women; certainty of evidence not reported)^8^. Again, for those who used epidurals during the second stage, the overall effect estimate of eight RCTs revealed no significant difference in caesarean section rates between upright and horizontal groups (RR=0.94; 95% CI: 0.61–1.46, I^2^=69%, tau^2^=0.13, p=0.07; 4316 women; very low certainty of evidence of effect)^22^.

### Subgroup analysis by position type

One SR included in the overview analyzed data according to position type^8^. This review found contradicting effect sizes between squatting using cushion, chair and stool compared with horizontal on duration of birth, assisted vaginal birth, during the second stage of labor. The cause of variations in the results of these outcome is not clear (Table 4).

## DISCUSSION

### Main findings

Three Cochrane SRs^8,9,22^ with a total of 18697 women were included. All the included SRs reported various comparators with the definitions and classifications of upright and horizontal positions, type of outcome measures, and the quality of RCTs within each SR. Women in the upright position with no epidural analgesia were more likely to experience a significantly shorter duration of the first stage of labor and a significantly shorter duration of the second stage of labor.

During the first stage of labor without epidural analgesia, women in upright positions showed a significant reduction in rates of caesarean section, need for epidural analgesia and admission to neonatal intensive care units. However, there were no significant differences between the two groups on the duration of the second stage of labor and assisted vaginal birth. The same SR found no difference between assuming an upright position compared with horizontal on the duration of the first stage of labor, assisted vaginal birth and caesarean section among women using epidural analgesia^9^.

During the second stage of labor without epidural analgesia, women in an upright position showed significant reduction in rates of assisted vaginal births^8^. However, no significant differences were found between the two groups on rates of caesarean section. During the second stage of labor with epidural analgesia, there was no significant difference in the overall effect for operative birth and duration of the second stage of labor^22^.

### Limitations

All three included SRs showed differences in the direction and magnitude of effect in individual studies on the duration of labor and birth, assisted vaginal birth and caesarean section. Two of the SRs rated these outcomes as very low-quality evidence, which implies spurious results. The variations in the definitions of birth positions together with performance and detection bias may have contributed to the high heterogeneity observed in some of the results. The confidence in the effect estimate is limited by inconsistences in the results of subgroup analyses by position type, mainly on the three different types of squatting position (cushion, chair, stool) during birth. This has opened up a renewed interest in evaluating the effectiveness of the squatting position compared with other positions during the second stage of labor. The SRs that were included used statistical techniques such as the random-effect model, subgroup analysis and sensitivity analysis to aid in the understanding of the causes of variation in the findings for different outcomes. However, all these measurements have limitations so that any association discovered may be spurious (e.g. subgroup comparisons are observational by nature)^26,27^. One way to explore and understand such variation is through the synthesis of qualitative research. Several authors have confirmed that the synthesis of qualitative evidence can help to explain the findings of effectiveness of reviews or studies by identifying contextual factors that can influence the use of interventions in healthcare^14,26,28-32^.

The precise definitions of each maternal position and their biomechanical characteristics were not assessed in a systematic manner in all the included SRs in this overview. There is a lack of sufficient detail on the optimal procedure, material, intensity, interval and duration, and frequency of the description of maternal position to make the findings applicable for replication in research and clinical practice. However, this information can be sought from the original published studies reporting the research findings other than in the SRs. Consequently, there is uncertainty over the specific components that are responsible for the measurement of the effect of one position relative to that of another that might influence birth outcomes during childbirth. In addition, a question remains over whether the effectiveness in RCTs can be replicated in research or clinical practice.

### Quality of the evidence from the SRs

The SRs that were included in this overview were classified as high quality using the R-AMSTAR tool. However, the overall quality of the 65 trials that were included in the SRs was variable: only 19 used adequate methods of randomization, 11 reported adequate allocation concealment, 40 showed a low risk of attrition bias and 24 showed a low risk of reporting bias. Moreover, the trials that were designated low-quality grade tended to report larger effects than the high-quality trials^33,34^, which undermines the overall evidence of the overview.

### Comparison of the findings of included SRs with existing guidelines

The three Cochrane SRs included in this overview have been used in the development of several international guidelines for the management of intrapartum care for healthy women and babies. These guidelines are provided by the World Health Organization (WHO)^35^, the National Institute for Health and Care Excellence (NICE)^36^, the Association of Women’s Health, Obstetric and Neonatal Nurses (AWHONN)^37^ and the Royal Australian and New Zealand College of Obstetricians and Gynaecologists (RANZCOG)^38^. This overview of the findings of the SRs is consistent with these current international guidelines, which specify that women in labor should be discouraged from lying supine or semi-supine during birth and should be encouraged to adopt any other position that they find comfortable. It has also been recommended that women in labor should avoid supine positions and instead assume a variety of upright positions that may be used in anticipation of slow labor, such as kneeling, squatting, sitting and/or standing^37^.

### Compliance with the protocol

It was decided to deviate from the pre-specified protocol for this overview by excluding non-Cochrane SRs and including only Cochrane SRs. Empirical evidence suggests that Cochrane SRs provide more rigorous evidence than non-Cochrane SRs because they follow Cochrane procedures, which appear to improve the quality of evidence^39,40^. Several authors of overviews of reviews have found it relevant to include only Cochrane reviews as they are judged to be at low risk of bias^18,41-44^.

## CONCLUSIONS

The findings of the overview of reviews suggest that there are some benefits compared with harm for women assuming an upright position in childbirth. However, the included SRs reported wide variations with little or no information on the precise definitions and optimal positioning during childbirth. Furthermore, there is uncertainty about how upright positions might work or the components that are responsible for having an effect on birth outcomes and whether effectiveness in RCTs can be replicated in clinical practice or research.

Uncertainty remains as to the effect of the use of upright compared with horizontal positions on the duration of labor and birth, and operative births. The extent of the effect of upright compared to that of horizontal positions necessitates accurate definitions of each position and of their maternal biomechanical consequences to enable a safe replication of these methods. Hence, until the influence of each birth position on birth outcomes is better understood with well-designed studies, women should be encouraged to give birth in whatever position they find comfortable.

## Data Availability

Data sharing is not applicable to this article as no new data were created.

## PROTOCOL REGISTRATION

The overview protocol can be found at https://www.crd.york.ac.uk/prospero/display_record.php?ID=CRD42014014255. This was first published on 14 October 2014 and updated on 26 February 2020.
